# Re-evaluating how charge transfer modifies the conformation of adsorbed molecules†
†Electronic supplementary information (ESI) available. See DOI: 10.1039/c8nr02237b


**DOI:** 10.1039/c8nr02237b

**Published:** 2018-07-27

**Authors:** P. J. Blowey, S. Velari, L. A. Rochford, D. A. Duncan, D. A. Warr, T.-L. Lee, A. De Vita, G. Costantini, D. P. Woodruff

**Affiliations:** a Physics Department , University of Warwick , Coventry CV4 7AL , UK . Email: D.P.Woodruff@warwick.ac.uk; b Diamond Light Source , Didcot , OX11 0DE , UK; c Dipartimento di Ingegneria e Architettura , Università degli Studi di Trieste , V. Valerio 10 , Trieste , Italy; d Department of Chemistry , University of Warwick , Coventry CV4 7AL , UK . Email: G.Costantini@warwick.ac.uk; e School of Chemistry , University of Birmingham , Edgbaston , Birmingham , B15 2TT , UK; f Department of Physics , King's College London , Strand , London , WC2R 2LS , UK

## Abstract

The archetypal electron acceptor molecule, TCNQ, is generally believed to become bent into an inverted bowl shape upon adsorption on the coinage metal surfaces on which it becomes negatively charged. New quantitative experimental structural measurements show that this is not the case for TCNQ on Ag(111). DFT calculations show that the inclusion of dispersion force corrections reduces not only the molecule-substrate layer spacing but also the degree of predicted molecular bonding. However, complete agreement between experimentally-determined and theoretically-predicted structural parameters is only achieved with the inclusion of Ag adatoms into the molecular layer, which is also the energetically favoured configuration. The results highlight the need for both experimental and theoretical quantitative structural methods to reliably understand similar metal–organic interfaces and highlight the need to re-evaluate some previously-investigated systems.

## Abstract 2

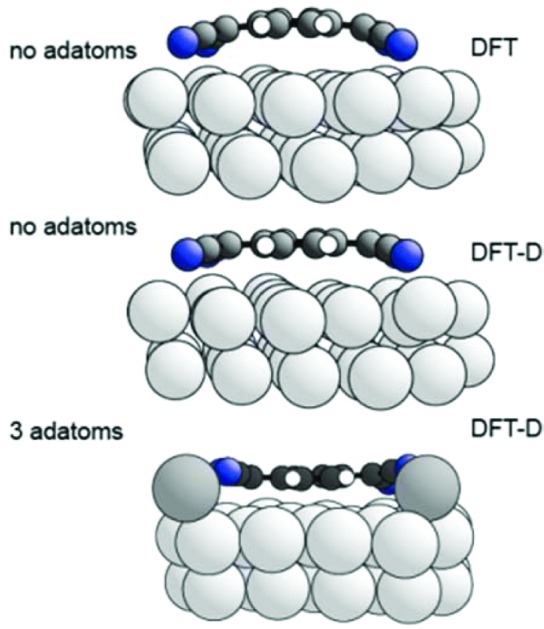
A combined quantitative experimental and theoretical structure determination shows TCNQ is not bent on Ag(111) as expected from conventional wisdom.

## 1. Introduction

It is now well-established that molecular adsorption on metal surfaces can lead to significant alterations in the electronic, chemical, and geometrical structure of both the adsorbed molecules and the underlying surface. Changes in the properties of strongly chemisorbed small molecules can play a key role in heterogeneous catalysis,^1^ while adsorbate-induced surface reconstructions are one clear manifestation of the influence of adsorbates on the surface.^2^ In the case of larger, essentially planar π-bonded molecules, of relevance to molecular electronics, the influence of the generally weaker adsorption on the molecular conformation is much less explored and most available structural information derives from studies based on a combination of density functional theory (DFT) and scanning tunnelling microscopy (STM); STM provides valuable information on lateral ordering but the details of the atomic coordinates that define the quantitative structure are often obtained only from the DFT calculations.

Here we illustrate the limitations of this approach for a system in which we find that only a combined experimental and theoretical investigation methodology that includes *quantitative* structural measurements is capable of solving the complexity of metal–organic interfaces involving π-bonded molecules. Specifically, we apply this methodology to an archetypal molecular adsorbate system, namely 7,7,8,8-tetracyanoquinodimethane (TCNQ) on a coinage metal surface, and show that the molecular conformation is significantly different from that accepted as conventional wisdom in the literature. This has arisen in part because of earlier failures to account for dispersion forces in DFT calculations but, more significantly, because quantitative experimental structural data highlight the need to account for adsorbate-induced substrate reconstruction in the calculations. Moreover, we demonstrate the importance of considering the coexistence of several energetically near-degenerate configurations in interpreting the experimental results.

The molecule TCNQ is a prototypical electron acceptor able to form organic charge transfer salts with high electron conductivity that have been influential in the development of organic photovoltaics,^3^,^4^ light-emitting diodes^5^,^6^ and field-effect transistor devices,^7^,^8^ and has been found to significantly reduce the hole injection barrier at interfaces with Cu and Ag surfaces.^9^,^10^ As a free molecule, the planar structure of TCNQ is very rigid due to the conjugated π-system that extends throughout the molecule, but if one or more electrons are transferred to it, becoming localised on the electron-withdrawing cyano groups, the central quinoid ring aromatises disrupting the π-conjugation.^11^ The peripheral carbon atoms thus become sp^3^ hybridised, rendering the molecule far more flexible. The results of essentially all published DFT calculations for TCNQ and its fluorinated analogue F4-TCNQ adsorbed on coinage metal surfaces predict a strong bending of the whole molecule with the cyano N atoms lying up to 1.4 Å below the C atoms of the quinoid ring while the cyano C atoms lie midway between the N atoms and the quinoid ring.^12^–^18^ Were this to be true, it would be a genuinely striking example of the influence on the molecular conformation of a nominally planar molecule by adsorption on a metal surface. Unfortunately, there is only fragmentary experimental structural information to support the suggestion. Specifically, near-edge X-ray absorption spectroscopy (NEXAFS) data from TCNQ on Cu(100) do indicate that the C–N bonds are tilted out of the plane of the central carbon ring^14^ but provide no evidence of bending of the overall carbon framework, nor do these results establish whether these tilted C–N bonds point down to the surface, out of the surface, or both.

Our experimental results for TCNQ adsorbed on Ag(111), using the normal incident X-ray standing wavefield (NIXSW) technique, show that there is no significant bending of the carbon framework of the molecule on this surface, although the N atoms do occupy at least two distinctly different heights on the surface, indicative of significant out-of-plane distortion of the cyano groups. Our dispersion-corrected DFT-D calculations predict that significant bending of the molecule must occur for adsorption on an unreconstructed surface, albeit of smaller amplitude than in calculations that take no account of dispersion forces. However, the DFT-D calculations reproduce the near-planar average geometry found experimentally, as well as the multiple N atom heights, for structural models that include Ag adatoms within the TCNQ ordered network. The theoretical analysis also demonstrates that several of these models are almost degenerate in energy, so their coexistence should be considered in order to interpret the NIXSW measurements correctly. These results highlight the need for both quantitative experimental structural information and DFT calculations to establish the true molecular structure but also demonstrate the need for careful interpretation of NIXSW data.

## 2. Experimental and computational methods

Experimental characterisation of the single-layer TCNQ adsorption phases formed by vacuum deposition of the molecule onto a clean Ag(111) surface at room temperature was undertaken by STM and low-current low energy electron diffraction (LEED) in an ultra-high vacuum (UHV) chamber at the University of Warwick, and by low-current LEED, soft X-ray photoelectron spectroscopy (SXPS) and NIXSW in the UHV end-station installed on beamline I09 of the Diamond Light Source storage ring. At both locations the single crystal Ag(111) substrate (cutting precision of 0.1°) was prepared *in situ* using cycles of sputtering with 1 keV Ar^+^ ions for 30 minutes followed by annealing to ∼500 °C for 30 minutes. A clean well-ordered sample was obtained as judged by LEED and STM at Warwick, and by LEED and SXPS at Diamond. LEED patterns obtained at both sites were used to provide a clear reference of the preparation of the same TCNQ adsorption phases (under closely similar preparation conditions) for the complementary STM and synchrotron-radiation based experiments. All STM images in this work were analysed, plane corrected and flattened using the Gwyddion open-source software.^19^

DFT calculations were performed for different model structures in the large unit mesh phase investigated using the plane-wave pseudopotential package QUANTUM ESPRESSO (QE)^20^ with ultrasoft pseudopotentials^21^ with an energy cutoff of 408 eV and a GGA-PBE^22^ exchange–correlation functional. Dispersion-corrected DFT-D calculations used the method proposed by Grimme^23^ as well as the vdW-DF method^24^ that are implemented in the QE package.^25^ A recent review of some 200 different density functionals provides a broad picture of the field,^26^ and remarks that these two functionals have proved popular in a range of applications in molecular interactions. However, molecular adsorption on metal surfaces presents some significantly different challenges,^27^ such as the role of screening, and while the DFT-D method has proved able to reproduce experimental molecule-substrate height measurements for a number of systems,^28^ the last few years have seen the development of more advanced functions specifically designed for these problems that are based on a more exact description of the underlying physics, with several reviews being published of the relative merits of these different approaches.^29^–^32^ Ultimately, the effectiveness of any of these different approaches can only be judged by comparison with experimental measurements of bonding distances and energies, and it is unclear to what extent demonstrated success with one system means the same method will prove to be optimal for a different system. Rather few experimental results for molecular adsorption on surfaces are available, and bonding distances have mostly been obtained from NIXSW experiments, the same experimental method that we have used here. Newer, more ‘ab initio’ approaches, including those proposed and refined successfully by Tkatchenko, Scheffler and co-workers, have been found to reproduce well not only experimental bonding distances, but also binding energies.^33^–^35^ By contrast, DFT-D has been found to significantly overestimate absolute binding energies, although bonding distances prove to be more reliable. In the present work the main structural results that we present have been obtained directly from experiment, so DFT calculations are performed only to provide some further insight into the interpretation of these results; for this purpose, calculations based on these earlier functionals may be expected to suffice. Our recent experience^36^ in studying related systems using the latest vdW^surf^ 33 functional leads us to expect no significant difference in the qualitative structural conclusions obtained here.

In view of the very large unit cell, *k*-point sampling was restricted to the *Γ*-point alone. The Ag(111) surface was modeled with three-layer repeated slabs, separated by a vacuum gap of ≈14 Å. Only the coordinates of atoms of the adsorbed molecules and the upper two Ag layers were allowed to relax.

## 3. Results and discussion

### 3.1 Adsorbate phase characterisation

**Fig. 1:**
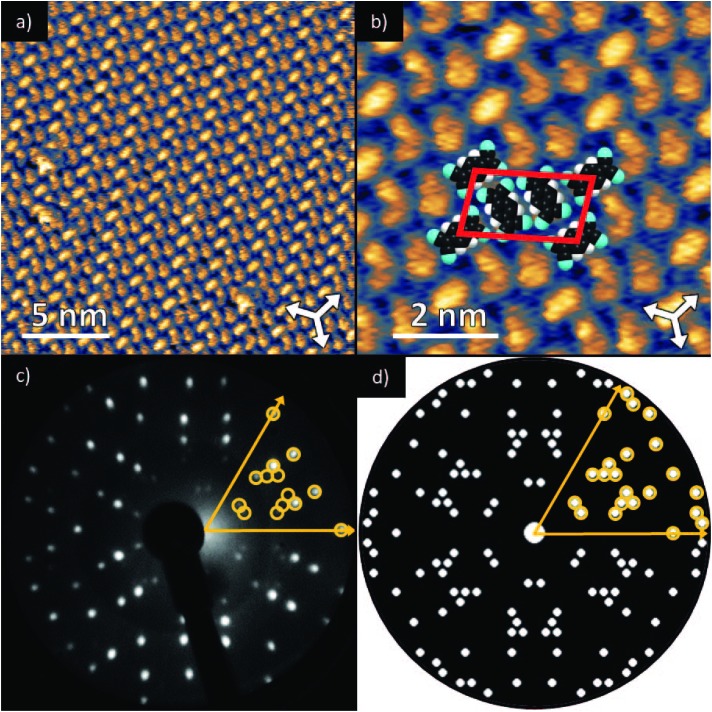
(a & b) STM images at two different magnifications of the 
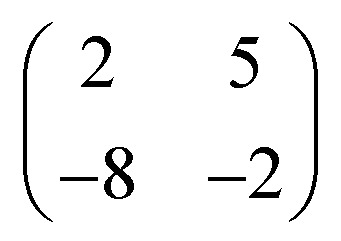
 ordered phase formed by TCNQ on Ag(111) (*V*_samp_ = –1.00 V, *I* = 55 pA). The substrate <110> directions are indicated by the white arrows. Superimposed on (b) is the surface unit mesh and a schematic representation of the TCNQ molecules. H atoms are coloured white, N atoms blue, C atoms black. For larger area STM images see ESI.† (c) LEED pattern recorded at a kinetic energy of 14.5 eV. The location of the beams match those predicted for a 
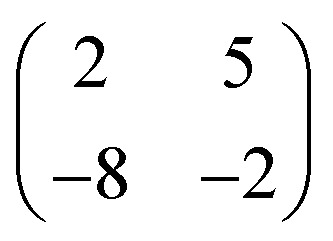
 commensurate matrix as shown in the simulated pattern (d) obtained using the LEEDpat program,^37^ including the beams from the 6 symmetry-equivalent domains arising from the 3*m* symmetry of the substrate. The predicted beams in one sector of (c) are superimposed as open rings on the experimental pattern in (b). Yellow arrows correspond to the <211> directions of the primitive translation vectors of the substrate unit mesh.

Deposition of TCNQ onto the Ag(111) surface by *in situ* vacuum evaporation led to the observation in both STM and LEED of two different ordered phases; initial deposition led to coexistence of these two phases, but each could be isolated by following different preparation conditions. Here we focus on the commensurate phase (that is accessible to DFT modelling) formed by annealing a saturation coverage to 550 K, or directly by depositing a submonolayer coverage at room temperature. (The existence of the second, incommensurate, phase has previously been reported by Wackerlin *et al.*^38^) STM of this phase (Fig. 1a) shows the TCNQ molecules as oblong protrusions that arrange into rows of ‘windmill’ units in which four molecules spiral around a central point. This ‘windmill’ motif is a feature common to many TCNQ adsorption structures on metal surfaces, both when deposited alone^12^,^15^,^39^ and coadsorbed with additional metal atoms.^15^,^38^,^40^–^43^ STM measurements indicate that this phase is commensurate to the underlying Ag(111) substrate with a unit mesh containing three TCNQ molecules, defined by net vectors 
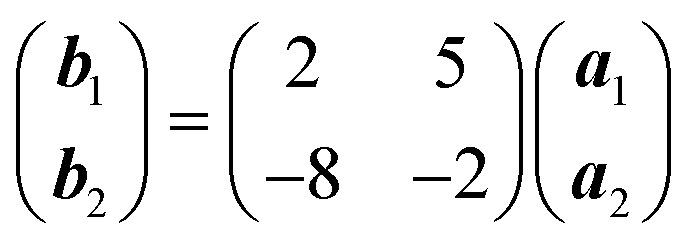
 where **a**_**1**_ and **a**_**2**_ are the primitive translation vectors of the Ag(111) surface. The simulated LEED pattern derived from this unit mesh (Fig. 1c) shows excellent agreement with the experimental LEED pattern (Fig. 1b), thus confirming, by a technique not susceptible to instrumental drift or calibration errors, the accuracy of the measured unit mesh.

**Fig. 2:**
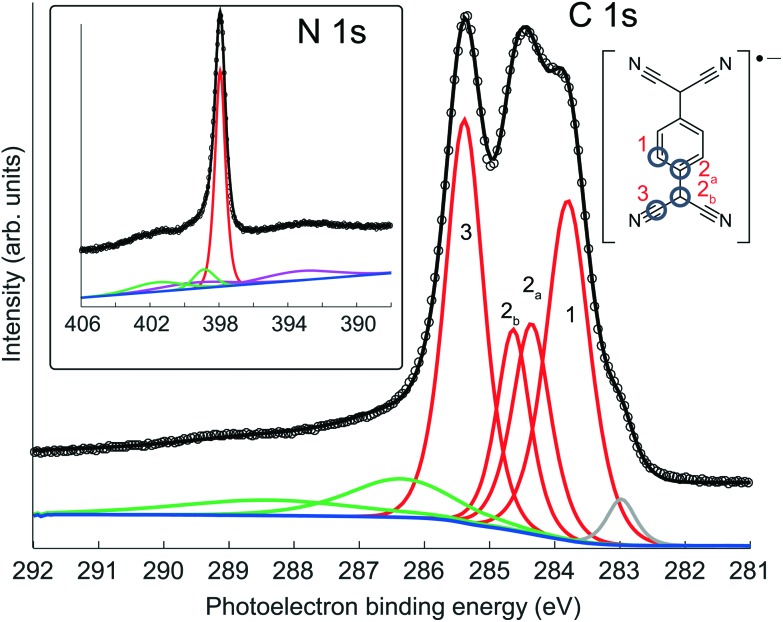
SXP C 1s and N 1s spectra obtained from TCNQ on Ag(111) at photon energies of 435 eV and 550 eV, respectively. The four-component fit (red) to the C 1s spectrum was constrained to the molecular stoichiometry. Also seen are lower kinetic energy shake-up satellites (green) and a small higher kinetic energy component (grey) associated with radiation damage. The N 1s peak has underlying plasmon losses (purple) from the Ag 3d emission peaks.

Fig. 2 shows high-resolution C 1s and N 1s SXP spectra obtained from this phase, with the binding energies of the main fitted components shown in Table 1. The C 1s spectrum clearly shows at least three distinct peaks, which have been fitted with four components corresponding to the four chemically inequivalent C species in TCNQ (see the inset of the C 1s spectrum); these four peaks were fitted allowing ±0.1 eV variation in their FWHM and the integrated areas were constrained to be within ±10% of their relative stoichiometry in the molecule. In previous reports, C 1s XPS has been used to deduce the charge state of TCNQ, with the relative binding energies and overall line shape of the spectrum being characteristically different when TCNQ is negatively charged compared to when it is neutral^38^,^44^,^45^ Based on this interpretation, the C 1s spectrum here is consistent with previous XPS measurements of negatively charged TCNQ,^38^,^43^–^45^ indicating that TCNQ accepts electrons from the Ag(111) substrate. The value of the N 1s binding energy is also in good agreement with other systems in which TCNQ is believed to be negatively charged.^38^,^43^–^45^ UPS measurements, reported both here and in a previous study,^38^ show a work function increase of 0.4 eV when TCNQ is deposited on clean Ag(111). This further reinforces that TCNQ does accept electrons from the Ag(111) substrate as neutral adsorbates would be expected to decrease the work function *via* the ‘push-back’ effect.^46^

**Table 1:** Measured SXPS binding energies of the main C 1s and N 1s components

Component	CH	CC_1_	CC_2_	CN	N
Binding energy/eV	283.9	284.4	284.6	285.4	397.9

### 3.2 NIXSW structural measurements

The NIXSW technique^47^,^48^ used here to obtain the structural information leads to parameter values that need to be interpreted carefully if reliable deductions are to be made regarding the true structure. In order to understand this, we outline here the key aspects of this method. NIXSW exploits the fact that, when an X-ray Bragg reflection is established in a crystal, the incident and reflected waves combine to form a standing wave with an intensity periodicity equal to that of the crystal scattering planes. Because of the strong backscattering out of the crystal there is a finite range of photon energy (X-ray wavelength) over which the standing wave is present, and within this range the phase of the standing wave relative to the crystal planes shifts in a systematic fashion. By monitoring the X-ray absorption at a particular atomic species as this standing wave is swept through the crystal and the region above its surface, the location of these atomic absorbers relative to the crystal scatterer planes can be determined. In the present case, the X-ray absorption at the C and N atoms of TCNQ was monitored when scanning through the (111) Bragg condition at normal incidence to the (111) surface by measuring the intensity variation of the 1s photoemission components, providing chemical-state selectivity in the NIXSW profiles from the locally-distinct constituent atoms. The photoemission variation obtained from such a photon energy scan can be uniquely fitted by two parameters, the coherent position, *P*, and the coherent fraction, *f*, after correcting for quadrupolar backward-forwards asymmetry in the photoemission.^49^ In the case of a single well-defined site of the photo-absorbing atoms, the value of *P* is a direct measure of the height, *D*, of this site above the crystal scatterer planes (*D* = (*P* + *n*)*d*_111_ where *d*_111_ is the interlayer spacing of the crystal scattering planes and *n* is an integer chosen to ensure that inter-atomic distances are physically reasonable). In this case *f* is effectively an order parameter that, for perfect static and dynamic order, would be equal to unity; in practice it is typically found to be ≥∼0.80. Lower values of *f* are generally not simply attributable to some arbitrary disorder, but indicate that two or more different heights must contribute to the measurement. For example, if two different sites with heights *z*_1_ and *z*_2_, relative to nearest extended bulk scattering plane, are equally occupied then (again assuming perfect static and dynamic order).

1 *f* exp(2π*D*/*d*) = (exp(2π*iz*_1_/*d*) + exp(2π*iz*_2_/*d*))/2

An important feature of this equation is that the value of *f* is sensitive to the difference in these heights; in particular, if the two heights differ by *d*/2, then the coherent fraction is zero despite the system being perfectly ordered.^50^ The more general expression for summing over multiple sites is given in the ESI.† These considerations are crucial to the proper interpretation of the parameters obtained from the NIXSW experiments, which, from our investigation, are listed in the top row of Table 2. Notice that although the coherent fraction values for all the C 1s components are close to unity (*f* ≥ 0.89), consistent with a single height of the absorbing atoms, the value of *f* for the N atoms is much lower (0.39), clearly indicating that the N atoms must occupy sites with at least two different heights. The heights of the different C atoms differ by no more than 0.10 Å, so the core of the molecule is *not* significantly bent (note that the lower spectral resolution of the photoemission spectra recorded in the higher photon energy NIXSW scans precludes separation of the CC_1_ and CC_2_ components). Moreover, the *D* value for the N atoms, which must represent a weighted average of the different contributing heights, is essentially identical to that of the C atoms in the C≡N moieties. However, the fact that the N atoms must occupy two significantly different sites is consistent with the idea that the negatively-charged adsorbed TCNQ is flexible, and is no longer rigidly planar.

**Table 2:** Coherent position (shown as *D* = (*P* + 1)*d*_111_) and coherent fraction values *f* obtained experimentally by NIXSW from the different C 1s photoemission components and from the N 1s emission (error estimates, discussed in ref. 43, are shown in parentheses in units of 0.01), compared with values obtained from DFT-D calculations for different structural models. The inequivalent C atom contributions are labelled as in Fig. 2. The total formation energies per unit mesh, relative to those of the model without adatoms, are shown in parentheses. For a representative set of NIXSW absorption profiles see ESI

	*F*				*D*/Å			
	CH	CC	CN	N	CH	CC	CN	N
**Experiment**	0.95(10)	0.99(10)	0.89(10)	0.39(10)	2.86(5)	2.78(5)	2.76(5)	2.75(5)
**DFT-D**								

Adatoms (Δ*E*/meV)								
None (0)	0.98	0.98	1.00	0.99	2.82	2.79	2.60	2.38
1 α (–101)	0.99	0.99	0.93	0.77	2.80	2.79	2.67	2.48
1 β (–46)	0.98	0.99	0.91	0.69	2.80	2.80	2.67	2.44
2 αβ (–111)	0.99	0.99	0.87	0.56	2.78	2.79	2.75	2.64
2 ββ (+4)	0.95	0.98	0.90	0.57	2.75	2.78	2.77	2.70
3 (–55)	0.98	0.99	0.92	0.66	2.74	2.78	2.84	2.88

Weighted average at RT	0.99	0.99	0.88	0.60	2.78	2.79	2.73	2.59

### 3.3 Structure interpretation and general discussion

The existence of at least two different N atoms heights (that may differ by as much as ∼0.9 Å according to eqn (1) in order to account for the measured coherent fraction) clearly indicates some asymmetry in the local coordination of the TCNQ molecule on the highly-symmetric Ag(111) surface. A way in which this might be achieved is through the presence of Ag adatoms within the ordered TCNQ network on the surface. Our STM images show no obvious features that could be attributed to adatoms, but similar absences have been reported in earlier studies of other systems^51^,^52^ and our own DFT-D simulated images based on the model, described below, which includes Ag adatoms, also shows no adatom-related image features (see ESI†). In the STM images of Fig. 1 the molecules are seen to arrange with negatively charged cyano groups in close proximity, which would be expected to create unfavourable electrostatic repulsions, were the adlayer to comprise only TCNQ molecules. Similar, seemingly unfavourable, assemblies have also been observed on Cu(111)^12^,^15^,^39^ and Cu(100),^14^ with the results of DFT calculations suggesting that the formation of a stress field, generated by the lifting of substrate atoms out of the surface plane by ∼0.3 Å, overcomes theses electrostatic repulsions.^12^,^15^ However, in contrast to the NIXSW results for the present system, these stress field models feature the TCNQ molecules in a considerably bent conformation, which is clearly not present here. An alternative way in which the observed ordering of TCNQ molecules might be rendered stable could be through the presence of Ag adatoms, incorporated into the molecular assembly, which might act as positive counterions and overcome the electrostatic repulsions. The general phenomenon of 2D metal–organic networks on surfaces is well known, and coadsorption of Mn, Fe or Ni atoms with TCNQ on Au(111),^17^ Cu(100),^14^,^53^ Cu(111)^15^ and Ag(100)^41^,^54^ is known to result in such networks. STM images have also been interpreted as indicating the presence of Au adatoms in an ordered F4-TCNQ structure on Au(111),^17^ in structures formed by TCNQ on Au(111) close to step edges and when co-adsorbed with an electron donor molecule;^18^ indeed, it has been previously suggested that Ag adatoms may also be present with TCNQ on Ag(111).^38^,^55^ Moreover, step-edge etching has been reported during adsorption of F4-TCNQ on Cu(100)^56^ and for the closely-related molecule, TCNE (tetracyanoethylene) on Ag(111), implying incorporation of metal adatoms into the resulting molecular networks may occur.^57^ In the case of the Mn + TCNQ/Cu(100) system, Shi *et al.*^53^ reported a DFT simulation of this structure that shows two distinctly different N heights above the surface, differing by 0.90 Å, with N atoms bonded to the Cu substrate lower than those bonded to the Mn adatoms. This system, albeit with chemically distinct metal adatoms, thus shows at least one of the key structural components that our NIXSW data identify as a feature of the Ag(111)-TCNQ system. However, in contrast to our NIXSW and DFT-D results for TCNQ/Ag(111), DFT calculations (without dispersion corrections) of the Mn + TCNQ/Cu(100) system indicate bent TCNQ molecules.

**Fig. 3:**
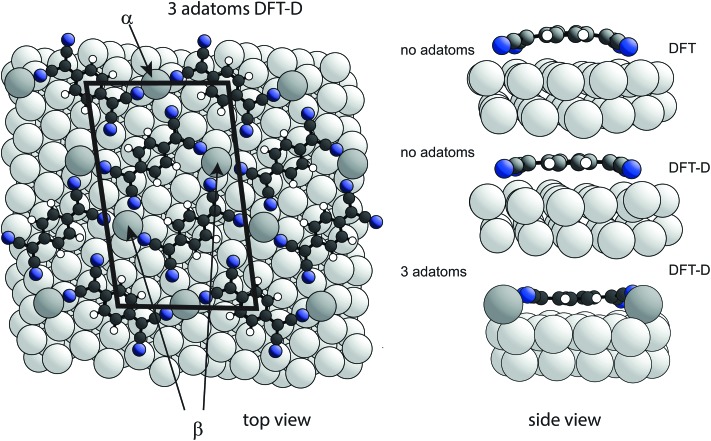
Left: Top view of the DFT-D-optimised structural model of the TCNQ surface phase with three Ag adatoms per unit mesh (shown by the black lines). Notice that there are two symmetrically distinct Ag adatom sites in this model, labelled α and β. Alternative models with 1, 2 or 3 Ag adatoms missing were also explored (Table 2). Right: Side views of a single molecule within the no-adatom model and in the 3-adatom model resulting from both DFT and DFT-D calculations. Ag adatoms are shaded darker than the substrate atoms. Other colours as in Fig. 1(a).

To explore the possible role of Ag adatoms in our system we have performed DFT calculations both with and without dispersion corrections for a number of models of the Ag(111)-TCNQ structure (Fig. 3). The results for the DFT-D calculations are summarised in Table 2. As expected, the inclusion of van der Waals forces in the DFT-D calculations for all models leads to molecular heights significantly (∼0.3–0.5 Å) lower than those given by calculations without dispersion corrections; these DFT-D values of the height of the molecule above the surface are much closer to the experimental values for all the structural models. In contrast, additional calculations using the alternative vdW-DF method^24^ yielded slight underbinding, *i.e.*, adsorbate heights larger (by ∼0.4 Å) than those obtained without dispersion corrections, in worse agreement with the experimental NIXSW data (for detailed results for these other functionals see ESI†). Our experimental measurements of the molecule-substrate layer spacing clearly provide a basis for identifying the DFT flavour that best describes the system. In the present case this is the DFT-D method, which also predicts significantly less molecular bending (Fig. 3). Models including adatoms clearly give much better agreement with the experimental structural parameter values. In the absence of Ag adatoms, DFT-D calculations predict some bending of the carbon core of the molecule, with the cyano C atoms 0.22 Å below those in the central ring, and a further downward bend of the C–N bonds by 0.22 Å. This degree of bending is much smaller than in previous calculations that take no account of dispersion forces^13^–^17^ and in our own dispersionless DFT calculations in which the height difference of the CN carbon atoms and the central ring is 0.58 Å. In all cases, the addition of an increasing number of Ag adatoms in the structure leads to a further flattening of the molecular shape, while a *distribution* of different N heights due to bonding to either adatoms or substrate atoms is predicted, leading to a reduction in the predicted coherent fraction for this species.

The relative energies of the different structures obtained in the DFT-D calculations, taking account of the different numbers of Ag atoms in the different models by using the bulk cohesive energy per atom as a reference level for adatom formation,^58^ are included in Table 1 and also favour most of the adatom models. Two models (1 adatom in the α site, and 2 adatoms – one each in the α and β sites – Fig. 3) have lower energies than the other models, but because the energy differences are only a few tens of meV one would expect co-occupation of several models at room temperature. Rather than comparing the experimental NIXSW data with a specific model, it is therefore more appropriate to describe the TCNQ surface phase on Ag(111) system as a canonical distribution of different adatom states in thermal equilibrium at room temperature. Using estimates of the relative occupation of the different structures based on Boltzmann factors with the energies in Table 1 and appropriate multiplicities, one can deduce the expected NIXSW parameter values for a weighted average of these occupations. These values are included in Table 1 and appear to be dominated by the two lowest energy structures, yielding a predicted 58% adatom site average occupancy at 300 K. An estimate of possible corrections to the relative occupations of different structures due to vibrational or slight off-equilibrium effects indicates that they do not change these results significantly (see ESI†).

Overall, the agreement between the predicted and experimental average layer spacings and coherent fractions is good. Discrepancies in the absolute heights are mostly less than 0.10 Å, and while the predicted *f* value for the N atoms is significantly higher than that measured experimentally, the predicted *f* values take no account of static and vibrational disorder for which a reduction in the coherent fraction of up to about 20% is generally found to be typical.^47^,^48^,^50^

Coincidentally, in the absence of adatoms, DFT-D calculations predict a significant (∼0.6 Å) buckling of Ag surface atoms, in particular for those atoms in close proximity to the molecular cyano groups. Essentially the same effect has been reported in several papers for DFT calculations for TCNQ or the closely-related TCNE molecule adsorbed on metal surfaces.^12^,^14^,^15^,^55^,^59^ The energy cost of this rumpling may be an added reason why adatom models are energetically favoured in our case, because the surface layer is significantly more planar when adatoms are included in the simulations. In this regard it is notable that DFT calculations (using the local density approximation (LDA) to the exchange–correlation energy functional – without dispersion corrections) for TCNE on Cu(100)^59^ were performed for model structures both with, and without, Cu adatoms. In fact the calculations indicate that the adatom model is significantly favoured energetically, yet the authors conclude that the unreconstructed rumpled surface model is more consistent with STM images. It would be interesting to revisit this system with more advanced computational methods as well as quantitative experimental structural measurements. Indeed, it is also notable that the possible role of Cu adatoms was not considered in the investigation of the Cu(100)/TCNQ system^14^ for which NEXAFS results indicated average tilt angles of the C–N bonds of ∼10°. This value is rather similar to the tilt angles found in our investigation of Ag(111)/TCNQ (∼11° pointing down to the surface and ∼7° pointing up out of the surface), so a similar twisted, rather than bent, TCNQ species on an adatom-modified structure may occur on Cu(100).

## 4. Conclusions

In summary, our combined experimental and theoretical structural study of TCNQ on Ag(111) indicates that although charge transfer to the molecule does occur, the carbon framework of the molecule is not bent; instead, only the cyano groups are twisted out the plane of the central carbon ring. Our theoretical calculations show that this is a direct consequence of the participation of Ag adatoms in the TCNQ surface phase. Compared to previous work in related systems, our results significantly improve the understanding of this prototypical metal–organic interface by combining four key factors: we have conducted a quantitative experimental determination of the structure, our DFT calculations take account of van der Waals forces, the choice of the DFT dispersion corrections was based on experimental data, and a canonical distribution of different configuration states in thermal equilibrium was used to describe the room temperature experiments.

As noted above, there have been previous suggestions (but no definitive proof) that substrate adatoms may be involved in the structures formed by TCNQ on Ag(111)^38^,^55^ but also for TCNQ,^18^ and F4-TCNQ^17^ on Au(111). Here, we clearly demonstrate that such adatoms are present with TCNQ on Ag(111). As adsorbed TCNQ molecules are reported to be negatively charged on Ag(111) in all these cases, the associated image charges will lead to strong dipoles that would be expected to repel each other. The inclusion of positively-charged metal atoms provides one way of stabilising the closed-packed structures observed, effectively creating a metal–organic charge-transfer salt, similarly to what occurs when transition^14^,^15^,^17^,^41^,^53^,^54^ or alkali metal atoms^38^,^40^,^42^,^43^ are intentionally co-deposited with TCNQ. How widespread might this phenomenon be? We speculate that the types of structures described here are quite common and, as molecule-substrate interactions are typically stronger on Cu surfaces than on Ag or Au, obvious candidates would be TCNQ on Cu(100) (discussed above), but also TCNQ or F4-TCNQ on Cu(111).^12^,^13^,^15^,^60^ In fact, a NIXSW investigation of the latter system has been published, but the coherent fractions reported (0.43, 0.28, 0.15 for F, N and C respectively) are so low that attributing the associated coherent positions of the F and N atoms to single heights, as reported in this paper,^13^ is highly questionable. The lack of information regarding the coverage or surface ordering in the surface studied makes it difficult to identify the origin of this problem, but it is tempting to speculate that the fact that the coherent fraction for the N atoms is significantly less than that for the F atoms might be consistent with multiple N heights and thus the influence of Cu adatoms. Evidently this and similar TCNQ/metal surface interfaces are systems that deserve a more thorough investigation.

## Conflicts of interest

All authors declare no conflicts of interest.

## Supplementary Material

Supplementary information

## References

[cit1] King D. A. and Woodruff D. P., Fundamental Studies of Heterogeneous Catalysis, Vol 4 of The Chemical Physics of Solid Surfaces, Elsevier, Amsterdam, 1982.

[cit2] King D. A. and Woodruff D. P., Phase Transitions and adsorbate restructuring at metal surfaces, Vol 7 of The Chemical Physics of Solid Surfaces, Elsevier, Amsterdam, 1994.

[cit3] Alves H., Pinto R. M., Maçôas E. S. (2013). Nat. Commun..

[cit4] Hsu C.-L., Lin C.-T., Huang J.-H., Chu C.-W., Wei K.-H., Li L.-J. (2012). ACS Nano.

[cit5] Zhou X., Blochwitz J., Pfeiffer M., Nollau A., Fritz T., Leo K. (2001). Adv. Funct. Mater..

[cit6] Blochwitz J., Pfeiffer M., Fritz T., Leo K. (1998). Appl. Phys. Lett..

[cit7] Menard E., Podzorov V., Hur S.-H., Gaur A., Gershenson M. E., Rogers J. A. (2004). Adv. Mater..

[cit8] Di C.-A., Yui G., Liu Y., Guo Y., Wu W., Wei D., Zhu D. (2008). Phys. Chem. Chem. Phys..

[cit9] Di C.-A., Liu Y., Yu G., Zhu D. (2009). Acc. Chem. Res..

[cit10] Guillain F., Endres J., Bourgeois L., Kahn A., Vignau L., Wantz G. (2016). ACS Appl. Mater. Interfaces.

[cit11] Milián B., Pou-Amérigo R., Viruela R., Ortí E. (2004). J. Mol. Struct. (THEOCHEM).

[cit12] Stradi D., Borca B., Barja S., Garnica M., Diaz C., Rodriguez-Garcia J. M., Alcami M., de Parga A. L. V., Miranda R., Martin F. (2016). RSC Adv..

[cit13] Romaner L., Heimel G., Brédas J.-L., Gerlach A., Schreiber F., Johnson R. L., Zegenhagen J., Duhm S., Koch N., Zojer E. (2007). Phys. Rev. Lett..

[cit14] Tseng T.-C., Urban C., Wang Y., Otero R., Tait S. L., Alcamí M., Écija D., Trelka M., Gallego J. M., Lin N., Konuma M., Starke U., Nefedov A., Langner A., Wöll C., Herranz M. Á., Martín F., Martín N., Kern K., Miranda R. (2010). Nat. Chem..

[cit15] Barja S., Stradi D., Borca B., Garnica M., Díaz C., Rodriguez-García J. M., Alcamí M., de Parga A. L. V., Martín F., Miranda R. (2013). J. Phys.: Condens. Matter.

[cit16] Martínez J. I., Abad E., Flores F., Ortega J. (2011). Phys. Status Solidi B.

[cit17] Faraggi M. N., Jiang N., Gonzalez-Lakunza N., Langner A., Stepanow S., Kern K., Arnau A. (2012). J. Phys.J. Phys.
Chem. CChem. C.

[cit18] Della Pia A., Riello M., Stassen D., Jones T. S., Bonifazi D., De Vita A., Costantini G. (2016). Nanoscale.

[cit19] Nečas D., Klapetek P. (2012). Cent. Eur. J. Phys..

[cit20] Giannozzi P., Baroni S., Bonini N., Calandra M., Car R., Cavazzoni C., Ceresoli D., Chiarotti G. L., Cococcioni M., Dabo I., Dal Corso A., de Gironcoli S., Fabris S., Fratesi G., Gebauer R., Gerstmann U., Gougoussis C., Kokalj A., Lazzeri M., Martin-Samos L., Marzari N., Mauri F., Mazzarello R., Paolini S., Pasquarello A., Paulatto L., Sbraccia C., Scandolo S., Sclauzero G., Seitsonen A. P., Smogunov A., Umari P., Wentzcovitch R. M. (2009). J. Phys.: Condens. Matter.

[cit21] Vanderbilt D. (1990). Phys. Rev. B: Condens. Matter Mater. Phys..

[cit22] Perdew J. P., Burke K., Ernzerhof M. (1996). Phys. Rev. Lett..

[cit23] Grimme S. (2006). J. Comput. Chem..

[cit24] Dion M., Rydberg H., Schröder E., Langreth D. C., Lundqvist B. I. (2004). Phys. Rev. Lett..

[cit25] Barone V., Casarin M., Forrer D., Pavone M., Sambi M., Vittadini A. (2008). J. Comput. Chem..

[cit26] Mardirossian N., Head-Gordon M. (2017). Mol. Phys..

[cit27] Mercurio G., McNellis E. R., Martin I., Hagen S., Leyssner F., Soubatch S., Meyer J., Wolf M., Tegeder P., Tautz F. S., Reuter K. (2010). Phys. Rev. Lett..

[cit28] Tonigold K., Gross A. (2010). J. Chem. Phys..

[cit29] Klimes J., Michealides A. (2012). J. Chem. Phys..

[cit30] Lee K., Berland K., Yoon M., Andersson S., Schröder E., Hyldgaard P., Lundqvist B. I. (2012). J. Phys.: Condens. Matter.

[cit31] Prates Ramalho J. P., Gomes J. R. B., Illas F. (2013). RSC Adv..

[cit32] Berland K., Cooper V. R., Lee K., Schröder E., Thonhaser T., Hyldgaard P., Lundqvist B. I. (2015). Rep. Prog. Phys..

[cit33] Ruiz V. G., Liu W., Zojer E., Scheffler M., Tkatchenko A. (2012). Phys. Rev. Lett..

[cit34] Liu W., Tkatchenko A., Scheffler M. (2014). Acc. Chem. Res..

[cit35] Maurer R. J., Ruiz V. G., Camarillo-Cisneros J., Liu W., Ferri N., Reuter K., Tkatchenko A. (2016). Prog. Surf. Sci..

[cit36] Reinhard Mauer, private communication

[cit37] Hermann K. E. and Van Hove M. A., LEEDpat (version 4.2), 2014, http://www.fhi-berlin.mpg.de/KHsoftware/LEEDpat/index.html.

[cit38] Wackerlin C., Iacovita C., Chylarecka D., Fesser P., Jung T. A., Ballav N. (2011). ChemComm.

[cit39] Kamna M. M., Graham T. M., Love J. C., Weiss P. S. (1998). Surf. Sci..

[cit40] Abdurakhmanova N., Floris A., Tseng T.-C., Comisso A., Stepanow S., De Vita A., Kern K. (2012). Nat. Commun..

[cit41] Tseng T.-C., Abdurakhmanova N., Stepanow S., Kern K. (2011). J. Phys. Chem. C.

[cit42] Umbach T. R., Fernández-Torrente I., Ruby M., Schulz F., Lotze C., Rurali R., Persson M., Pascual J. I., Franke K. J. (2013). New J. Phys..

[cit43] Blowey P. J., Rochford L. A., Duncan D. A., Warr D. A., Lee T.-L., Woodruff D. P., Costantini G. (2017). Faraday Discuss..

[cit44] Precht R., Hausbrand R., Jaegermann W. (2015). Phys. Chem. Chem. Phys..

[cit45] Precht R., Stolz S., Mankel E., Mayer T., Jaegermann W., Hausbrand R. (2016). Phys. Chem. Chem. Phys..

[cit46] Braun S., Salaneck W. R., Fahlman M. (2009). Adv. Mater..

[cit47] Woodruff D. P. (1998). Prog. Surf. Sci..

[cit48] Woodruff D. P. (2005). Rep. Prog. Phys..

[cit49] Lee J., Fisher C., Woodruff D. P., Roper M. G., Jones R. G., Cowie B. C. C. (2001). Surf. Sci..

[cit50] Woodruff D. P., Cowie B. C. C., Ettema A. R. H. F. (1994). J. Phys.: Condens. Matter.

[cit51] Classen T., Fratesi G., Costantini G., Fabris S., Stadler F. L., Kim C., de Gironcoli S., Baroni S., Kern K. (2005). Angew. Chem., Int. Ed..

[cit52] Feng Z., Velari S., Cossaro A., Castellarin-Cudia C., Verdini A., Vesselli E., Dri C., Peressi M., De Vita A., Comelli G. (2015). ACS Nano.

[cit53] Shi X. Q., Lin C., Minot C., Tseng T.-C., Tait S. L., Lin N., Zhang R. Q., Kern K., Cerda J. I., Van Hove M. A. (2010). J. Phys. Chem. C.

[cit54] Feyer V., Graus M., Nigge P., Zamborlini G., Acres R. G., Schöll A., Reinert F., Schneider C. M. (2015). J. Electron Spectrosc. Relat. Phenom..

[cit55] Rodriguez-Fernandez J., PhD Thesis, Universidad Autónoma de Madrid, 2014.

[cit56] Katayama T., Mukai K., Yoshimoto S., Yoshinobu J. (2011). Phys. Rev. B: Condens. Matter Mater. Phys..

[cit57] Rodríguez-Fernández J., Lauwaet K., Herranz M. Á., Martín N., Gallego J. M., Miranda R., Otero R. (2015). J. Chem. Phys..

[cit58] Scheffler M. and Stampfl C., in Handbook of Surface Science, Vol. 2: Electronic Structure, ed. K. Horn and M. Scheffler, Elsevier, Amsterdam, 1999.

[cit59] Bedwani S., Wegner D., Crommie M. F., Rochefort A. (2008). Phys. Rev. Lett..

[cit60] Otero R., Gallego J. M., de Parga A. L. V., Martín N., Miranda R. (2011). Adv. Mater..

