# Supplementary material for: Re-evaluating how charge transfer modifies the conformation of adsorbed molecules
Source: Nanoscale. 2018 Jul 27;10(31):14984–92. doi: 10.1039/c8nr02237b (PMC6088372; doi:10.1039/c8nr02237b)
Supplement: Supplementary file 1 [file NR-010-C8NR02237B-s001.pdf]

Electronic Supplementary Material (ESI) for Nanoscale.
This journal is © The Royal Society of Chemistry 2018

Re-evaluating how charge transfer modifies the conformation of adsorbed molecules.

P.J. Blowey[1,2], S. Velari[3], L.A. Rochford[4,5], D.A. Duncan[2], D.A. Warr[4], T.-L. Lee[2], A. De Vita[3,6], G. Costantini[4], D.P. Woodruff[1]

[1]Physics Department, University of Warwick, Coventry CV4 7AL, UK

[2]Diamond Light Source, Didcot, OX11 0DE, UK

[3]Dipartimento di Ingegneria e Architettura, Università degli Studi di Trieste, V. Valerio 10 Trieste, Italy

[4]Department of Chemistry, University of Warwick, Coventry CV4 7AL, UK

[5]School of Chemistry, University of Birmingham, Edgbaston, Birmingham, B15 2TT, UK

[6]Department of Physics, King's College London, Strand London, WC2R 2LS, UK

STM

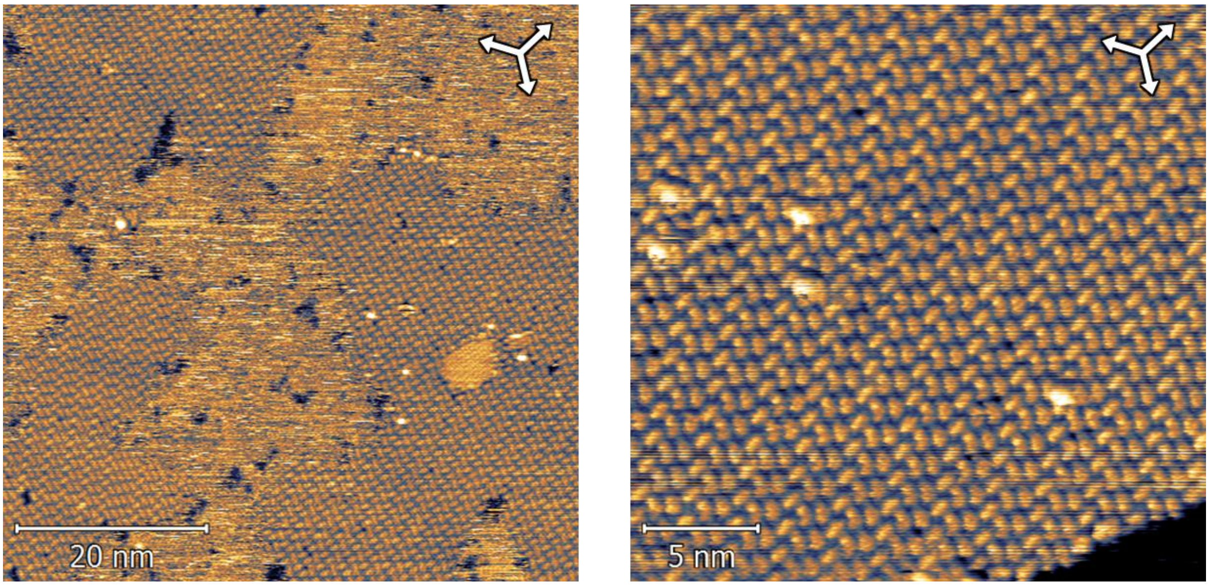

Figure S1*- Representative STM images of the prepared TCNQ Ag(111) adsorption phase. Left – A large area STM image showing three ordered molecular islands, recorded with scanning parameters of: –0.15 V, 100 pA. Right – STM image of a single TCNQ domain. A selected area of this image is presented in Figure 1 of the main paper. Scanning parameters: –1.0 V, 55 pA. In both images, the substrate <110> directions are indicated by the white arrows.*

Fig. S1 shows larger area STM images than that shown in Fig. 1(a) of the main paper. Analysis of multiple STM images suggests that the TCNQ adsorption phase is described by a unit mesh containing three TCNQ molecules with vectors of length $b_1 = (12.3 \pm 0.3)$ Å and $b_2 = (20.4 \pm 0.7)$ Å with an included angle of $(97 \pm 2)°$ and with vector $\mathbf{b_1}$ offset by $(24 \pm 2)°$ from the substrate <110> directions. These STM measured unit mesh parameters are consistent

with a commensurate surface structure described by a matrix of $\begin{pmatrix} 2 & 5 \\ -8 & -2 \end{pmatrix}$ (b_1 = 12.6 Å, b_2 = 20.8 Å, included angle of 97.3°, $\mathbf{b_1}$ offset by 23.4° from the substrate <110> directions) which is in excellent agreement with the experimentally observed LEED pattern presented in Figure 1 of the main paper.

As outlined in the main paper, an issue of interest is whether there is any evidence of features in the STM images that can be attributed to the location of the Ag adatoms. Figure S2 shows a comparison of simulated STM images obtained, using the Tersoff-Hamann approach [1], from the no-adatom (a) and the 3-adatom (c) DFT-D models with the experimental image (b) shown on the same scale. In addition a slightly larger area of the simulated image for the 3-adatom model, overlaid with the molecular model, is shown in panel (d), compared with a similar area experimental image that has been constructed by averaging over many unit meshes of the raw experimental image produced using the WSxM software package [2]. Notice that although the simulated images with and without adatoms do appear rather different in detail, no visible features are attributable to the location of the Ag adatoms. Indeed, the location of some of the Ag adatoms lie midway between two features of the simulated image that might otherwise have been attributed to adatoms. Moreover, while the unit mesh averaged image greatly reduces the ‘noise’ of the raw experimental image, only features attributable to the TCNQ molecules are visible. The fact that adatoms in metal-organic structures at surfaces might not be visible in STM is not surprising and has been reported previously (e.g. [3, 4]).

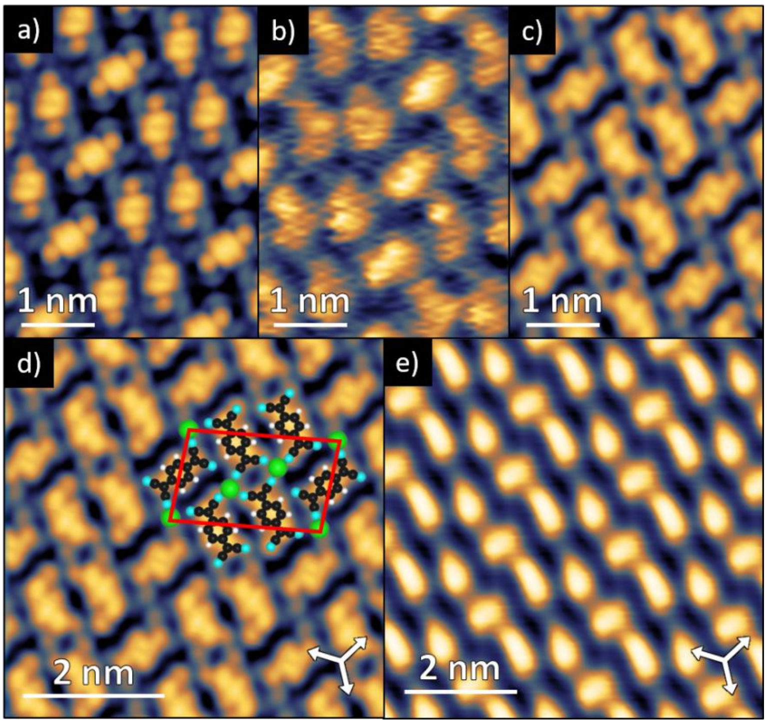

Figure S2- *(a) and (c) show DFT-D simulated images of the no-adatom and 3-adatom models described in the main paper and are compared with a similar area experimental image (b). (e) shows a unit-mesh-averaged experimental image, produced using the WSxM software package [2], compared with (d) the DFT-D simulation for the 3-adatom with the structural model overlaid.*

<u>NIXSW</u>

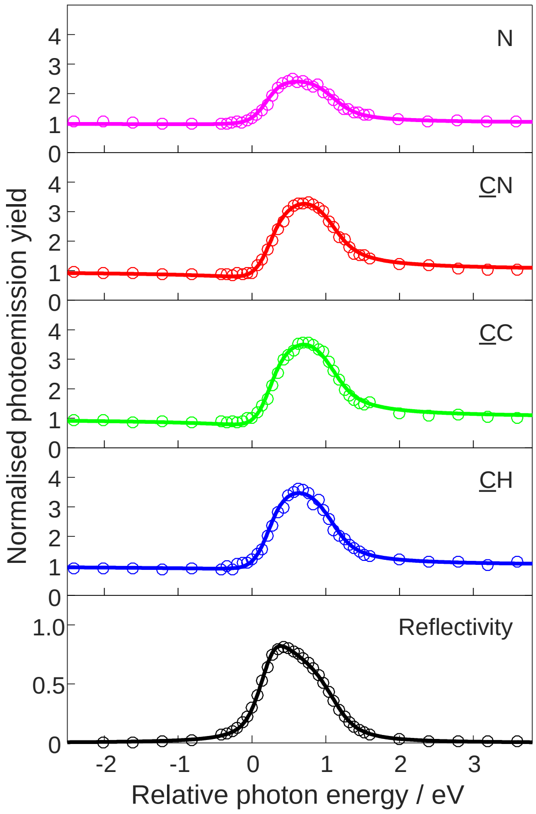

Figure S3 *– Sample set of NIXSW photoemission yield curves obtained using the [111] reflection for C and N in the TCNQ adsorption phase on Ag(111). Photon energies are quoted relative to the Bragg energy of 2.630 keV. Least square fits (solid lines) to the photoemission yields (circles) were obtained to extract the coherent fractions and coherent positions.*

The resulting experimental NIXSW structural parameters are listed in Table 2 of the main paper but also displayed in an Argand diagram representation in Figure S4.

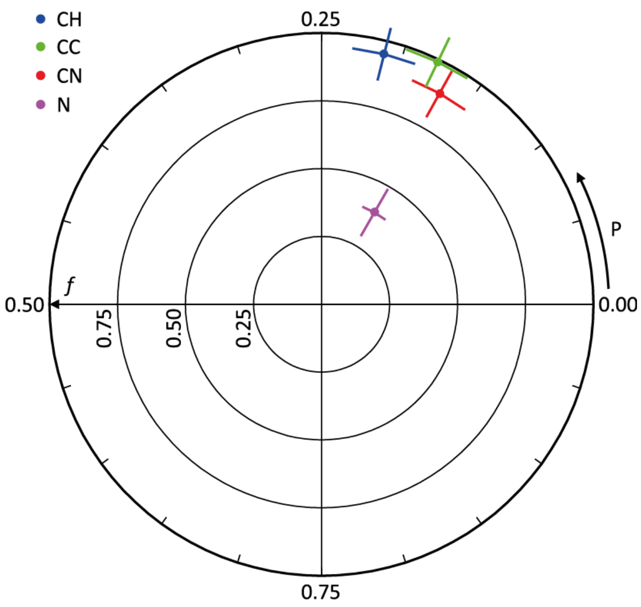

***Figure S4**. Experimental NIXSW structural parameter values shown in an Argand diagram representation*

<u>Predicted NIXSW parameter values obtained from the DFT calculations without dispersion correction and using the vdW-DF method</u>

	f				***D* / Å**			
**DFT**								
Adatoms	CH	CC	CN	N	CH	CC	CN	N
none (0)	0.96	0.94	0.99	0.98	3.30	3.12	2.72	2.38
1 α	0.95	0.94	0.79	0.52	3.29	3.15	2.83	2.44
1 β	0.96	0.94	0.81	0.59	3.34	3.20	2.84	2.42
2 αβ	0.99	0.97	0.73	0.23	3.31	3.21	2.98	2.68
2 ββ	0.93	0.97	0.78	0.36	3.39	3.27	2.97	2.52
3	0.87	0.96	0.71	0.12	3.45	3.36	3.13	2.88
**vdW-DF**								
Adatoms	CH	CC	CN	N	CH	CC	CN	N
none (0)	0.99	0.96	0.98	0.94	3.45	3.29	2.93	2.61
1 α	0.97	0.94	0.78	0.49	3.47	3.35	3.06	2.69
1 β	0.99	0.96	0.81	0.53	3.46	3.34	3.02	2.62
2 αβ	0.99	0.97	0.75	0.20	3.47	3.39	3.20	3.04
2 ββ	0.98	0.96	0.73	0.31	3.53	3.44	3.20	3.00
3	0.98	0.99	0.82	0.46	3.53	3.48	3.35	3.30

***Table S1** – Expected coherent fractions (f) and coherent positions (D, taken relative to the average height of the outermost Ag layer) obtained for the pure DFT and vdW-DF dispersion corrected structural models.*

To calculate the expected NIXSW parameters shown for the DFT and vdW-DF calculations in Table S1 and the DFT-D calculations presented in Table 1 of the main paper, the following equation was used:

$$f exp\left(\frac{2\pi i D}{d_H}\right) = \frac{1}{n}\sum_{j=1}^{n} f_j exp\left(\frac{2\pi i z_j}{d_H}\right)$$

where f is the overall coherent fraction, D is the overall coherent position in Å, d_H is the Ag(111) lattice spacing (2.359 Å), n is the total number of atoms considered within a single NIXSW component, f_j is the coherent fraction of atom j (here taken to equal 1, i.e. no inclusion of a Debye-Waller factor to account for thermal vibrations) and z_j is the height of atom j taken relative to the average height of the outermost Ag(111) layer in the model. Performing the summation on the right-hand side of the equation yields a complex number with a magnitude equal to f and phase angle equal to $\left(\frac{2\pi D}{d_H}\right)$, from which the fractional coherent position $\left(\frac{D}{d_H}\right)$ can be extracted. In NIXSW experiments, the coherent position corresponds to the distance of the absorber from the nearest scattering plane, which includes the projections of bulk scattering planes that extend beyond the substrate surface. This means that to obtain adsorption heights, any surface relaxations must be considered as they cause the surface layer to deviate away from the projected bulk scattering planes. For Ag(111), the surface relaxations are minimal with negligible deviation from the bulk-terminated structure [5, 6]. The coherent position can therefore be converted into an adsorption height by adding an appropriate number of Ag(111) lattice spacings (in this case 1). The above procedure was performed for each of the calculated structural models using the atomic coordinates, treating the atoms from the different NIXSW components (CH, CC, CN and N as identified by XPS, Figure 2 of the main paper) separately.

Possible impact of free energy and off-equilibrium effects.

We note that, apart from the approximations involved in the DFT treatment, the DFT prediction for the RT weighted average structure in Table 2 of the main paper neglects any vibrational contribution, because formation energies instead of formation free energies were used to construct the Boltzmann factors. We have also assumed that perfect equilibrium has been achieved at the time of the measurements [7]. The energy corrections required to account for these effects are expected to be of the order of k_BT (~25 meV) per adatom. Furthermore, both effects are expected to slightly stabilise the adatom structures. This is because in the surface structures of Fig. 3 (main text) the adatoms are relatively undercoordinated and thus freer to vibrate than in the reference (bulk, or straight step kink site [7]) configurations, and, independently, because of the slightly higher availability of adatoms occurring at all times before full thermal equilibrium is established.

A simple and effective way to estimate these effects is to recast them both by an effective correction $\Delta\mu_{corr}$ to the reference chemical potential for forming adatoms in the analysed structures. Rather than predicting this theoretically, we explore here the consequences of treating this upshift as a free parameter and then use the experimental NIXSW structural data to determine its best fit value. A plot of the relative standard error in the prediction of the structural data (evaluated for simplicity on all coherent positions and fractions and normalised to $\Delta\mu_{corr} = 0$) as a function of $\Delta\mu_{corr}$ is provided in Fig. S5, giving a small positive best fit value of $\Delta\mu_{corr} = 60$ meV. This slightly improves the predicted structural parameters of Table 1 (main text) to the new values shown in Table S2 below. This correction also yields an increase of the average occupation of adatom sites per unit mesh of the molecular adlayer, from 58% to 77%. Both values are consistent with a picture of partially occupied adatom sites in the investigated TCNQ Ag(111) adsorption phase, comparable to what was observed in ref. [8] where a 29% presence of adatom vacancies (i.e. 71% adatom site occupancy) was reported based on STM observations.

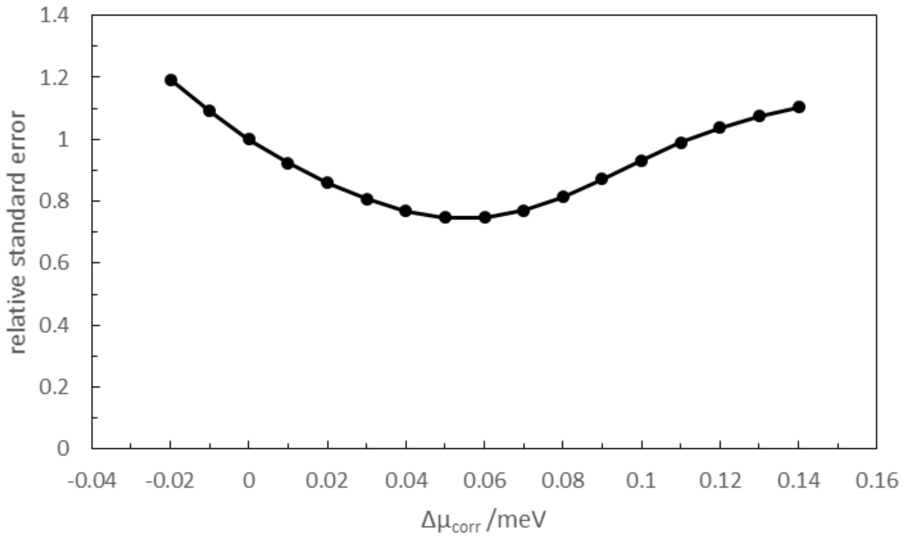

Figure S5 *– Plot of the relative standard error in the prediction of the structural data as a function of the effective correction* $\Delta\mu_{corr}$ *to the reference chemical potential for forming adatoms in the analysed structures.*

	F				*D* / Å			
	CH	CC	CN	N	CH	CC	CN	N
**Experiment**	0.95(10)	0.99(10)	0.89(10)	0.39(10)	2.86(5)	2.78(5)	2.76(5)	2.75(5)
**DFT-D**								
Avg at 300K (uncorrected, $\Delta\mu_{corr}$ = 0 meV)	0.99	0.99	0.88	0.60	2.78	2.79	2.73	2.59
Avg at 300K (corrected, $\Delta\mu_{corr}$ = 60 meV)	0.98	0.99	0.88	0.57	2.76	2.78	2.77	2.72

Table S2 *– Expected coherent fractions (f) and coherent positions (D), obtained from the corrected DFT-D results in comparison with the experimental values.*

1 J. Tersoff and D. R. Hamann, *Phys. Rev. B*, 1985, **31,** 805-813.

2 I. Horcas, R. Fernández, J.M. Gómez-Rodríguez, J. Colchero, J. Gómez-Herrero and A. M. Baró. *Rev. Sci. Instrum.*, 2007, **78**, 013705.

3 T. Classen, G. Fratesi, G. Costantini, S. Fabris, F.L. Stadler, C. Kim, S. de Gironcoli, S. Baroni and K. Kern, *Angew. Chem. Int. Ed.*, 2005, **44,** 6142-6145.

4 Z. Feng, S. Velari, A. Cossaro, C. Castellarin-Cudia, A. Verdini, E. Vesselli, C. Dri, M. Peressi, A. De Vita and G. Comelli, *ACS Nano*, 2015, **9**, 8697-8709.

5 E. A. Soares, V. B. Nascimento, V. E. de Carvalho, C. M. C. de Castilho, A. V. de Carvalho, R. Toomes and D. P. Woodruff, *Surf. Sci.*, 1999, **419**, 89-96.

6 P. Statiris, H. C. Lu and T. Gustafsson, *Phys. Rev. Lett.*, 1994, **72**, 3574-3577.

7 M. Scheffler and C. Stampfl, in: *Handbook of Surface Science, Vol. 2: Electronic Structure*, ed. K. Horn and M. Scheffler. Elsevier, Amsterdam 1999.

8 C. Wackerlin, C. Iacovita, D. Chylarecka, P. Fesser, T. A. Jung and N. Ballav, *ChemComm.*, 2011, **47**, 9146.
